# Breastfeeding and workplace support in Nepal

**DOI:** 10.1371/journal.pgph.0005059

**Published:** 2025-08-14

**Authors:** Mallika Shrestha, Saroj Adhikari, Nishant Shrestha, Gayatri Budha Magar, Babu Ram Pokhrel

**Affiliations:** 1 Health Office Parsa, Ministry of Health and Population, Kathmandu, Nepal; 2 Department of Public Health, Purbanchal University School of Health Sciences, Morang, Nepal; 3 Department of Health Sciences, Fanshawe College, London, Canada; St John's Medical College, INDIA

## Abstract

Working mothers encounter numerous challenges in exclusively breastfeeding their children despite overwhelming evidence of its benefits. This study examines exclusive breastfeeding (EBF) practices among employed mothers in Nepal, focusing on workplace support factors including maternity leave duration, flexible work schedules, task adjustments, breastfeeding policies, availability of lactation rooms, and dissemination of organizational support information. A cross-sectional study was conducted in Nepal among employed mothers with children under two years old. A self-administered online questionnaire was used to gather data from 201 mothers, and the results were analyzed using SPSS 16. EBF prevalence among employed mothers was 37.8%, with 76.4% identifying their return to work as the primary reason for discontinuing EBF. Only 18.9% of workplaces had designated rooms for breastfeeding, but such spaces increased the likelihood of EBF by 11.6 times (COR: 11.6; 95% CI: 4.7 – 28.2). Furthermore, task adjustment increased EBF by four times (COR: 4.2; 95% CI: 2.2 – 7.8). Maternity leave, combined with breastfeeding-friendly workplaces, significantly promotes EBF practices among working mothers in Nepal.

## Introduction

Nepal’s evolving labor landscape presents significant workplace challenges for women, particularly those employed in the formal sector regarding maternal and child health protections. Over the past decade, Nepal has experienced increasing female labor force participation across both formal and informal sectors. According to the World Bank’s gender data, the labor force participation rate for women stands at 28.7%, which remains substantially lower compared to men (53.9%) [1]. While 78% of women work in informal sectors, women in formal employment face distinct workplace challenges related to structured organizational policies and institutional support systems for maternal health.

The formal sector workplace environment for employed mothers in Nepal reveals critical gaps in organizational support infrastructure despite having more structured employment frameworks than informal sectors. Women employed in formal organizations—including government offices, hospitals, banks, educational institutions, and private companies—face unique challenges in balancing professional responsibilities with maternal health needs. The majority of these formally employed mothers lack adequate workplace support for breastfeeding, including insufficient access to dedicated lactation facilities, limited flexible work arrangements, and inadequate organizational accommodation for maternal responsibilities [2]. These deficiencies reflect deeper systemic gender inequities in how formal organizations structure workplace policies and distribute care responsibilities. A study by Bhandari et al., 2019 among women working in tertiary level hospitals in Nepal specifically highlighted that engagement in formal sector jobs was a primary barrier to exclusive breastfeeding (EBF) [3]. Similarly, Singh et al., 2024 demonstrated that maternal employment status, particularly in formal sector positions, significantly impacted EBF practices in Nepal, alongside factors such as antenatal care visits and poverty status [4].

The workplace-related challenges become particularly pronounced when examining Nepal’s maternity protection framework. While Nepal’s labor laws guarantee 98 days of maternity leave—of which only 60 days are fully paid—this provision falls short of international standards and creates additional financial pressures for working mothers [5]. The International Labour Organization (ILO) Maternity Protection Convention, 2000 (No. 183) and Recommendation No. 191 advocate for at least 14–18 weeks of paid maternity leave and comprehensive workplace breastfeeding accommodations [6]. More critically, Nepal’s current maternity leave structure contradicts recommendations from the World Health Organization (WHO) which call for six months of exclusive breastfeeding.

These workplace limitations have measurable consequences for maternal and child health outcomes. The importance of addressing these challenges is underscored by the substantial health benefits of breastfeeding: children who are breastfed have lower risks of developing asthma, obesity, type 1 diabetes, respiratory infections, ear infections, and gastrointestinal diseases [7], while mothers experience reduced likelihood of breast cancer, ovarian cancer, type 2 diabetes, and high blood pressure [8]. In low- and middle-income countries like Nepal, exclusive breastfeeding is associated with significant reductions in infant mortality, with exclusively breastfed infants facing only a 12% risk of death compared to three to four times higher mortality risk for non-exclusively breastfed infants [9]. Research from Nepal specifically shows higher child survival rates among children under 3 years of age whose mothers continued breastfeeding [10].

The formal sector employment context makes these workplace-related challenges particularly complex, as formally employed mothers often have structured work schedules, professional responsibilities, and organizational hierarchies that can either support or hinder breastfeeding practices [11]. Unlike informal sector workers who may have more flexibility in their work arrangements, formal sector employees face institutional constraints that require organizational policy interventions [12]. The workplace barriers facing formally employed Nepalese women have contributed to declining breastfeeding rates despite global recommendations and the theoretical advantages of formal employment protections. According to the 2022 Nepal Demographic and Health Survey (NDHS), exclusive breastfeeding rates declined to 56% from 70% in 2011 [13], a concerning trend that affects mothers across all employment sectors. This decline occurs against a global backdrop where only 48% of infants are exclusively breastfed according to UNICEF’s 2023 report [14], indicating that formal sector workplace-related challenges may be contributing to this critical health issue.

Several maternal factors compound these workplace challenges, with employment-related barriers intersecting with demographic and socioeconomic factors. Maternal age plays a significant role, with mothers who exclusively breastfed being notably older (average age 27.8 ± 5.2 years) compared to those practicing partial breastfeeding (23.9 ± 4.3 years). Additionally, maternal education level and marital status influence EBF practices [15], suggesting that workplace policies must account for diverse demographic profiles among working mothers.

Despite the existence of formal sector workplace laws in Nepal that theoretically support breastfeeding mothers—including the 98-day maternity leave provision and recommendations for necessary breastfeeding arrangements [16]—there remains a significant implementation gap within formal organizations. Limited evidence exists regarding the extent to which formally employed mothers receive adequate organizational support such as flexible working hours, designated breastfeeding areas, or access to workplace childcare facilities. This implementation gap is particularly concerning in formal sector environments where organizational policies and institutional support systems should theoretically provide better protection for working mothers compared to informal employment settings.

This study aims to address these formal sector workplace-related challenges by exploring the prevalence of exclusive breastfeeding among mothers employed in formal sector organizations in Nepal, adhering to WHO’s definition of EBF for the first six months of life. It evaluates the role of formal workplace support systems, including designated breastfeeding spaces, task adjustments, and flexible work hours, in promoting EBF among formally employed mothers. Given that maternity leave policies in Nepal offer a minimum of 98 days (approximately 3 months) and should be more readily implemented in formal sector organizations, this research investigates how current formal sector workplace practices align with both national and international breastfeeding recommendations. The findings will provide critical insights into the effectiveness of formal sector workplace policies in promoting breastfeeding and inform the development of targeted organizational interventions to improve working conditions for mothers while enhancing maternal and child health outcomes among Nepal’s formally employed women.

## Materials and methods

### Ethics statement

Ethical clearance was obtained from the Institutional Review Committee (IRC) of Purbanchal University School of Health Sciences (IRC ref no. 0022-080/81). Informed consent was sought from all participants through the online form. Participants who declined consent were automatically excluded from further access to the questionnaire. To ensure data confidentiality and privacy, several measures were implemented throughout the study. All responses were anonymized during data collection, with identifying information removed prior to analysis. Data were collected through a secure online platform (Google Forms), which required access via a unique survey link, preventing unauthorized submissions.

The dataset was stored on password-protected servers, accessible only to the principal investigator and authorized research team members. Additionally, all data were encrypted during transfer and storage, ensuring compliance with ethical standards for data protection and participation was entirely voluntary.

### Study design and area

A cross-sectional analytical study was conducted to assess exclusive breastfeeding practices among formally employed mothers in Nepal. The study utilized a self-administered questionnaire delivered through google survey tools to collect data from employed mothers with children between 6 months and under two years of age. The online platform allowed for broad geographic coverage, minimizing potential bias from face-to-face interviews and increasing the likelihood of receiving candid responses from participants.

### Study population and sampling

The target population included employed mothers with children under two years of age, working in any organizations across Nepal. A voluntary response sampling, a type of convenience sampling [17], was used to reach the desired participants. To minimize bias, the questionnaire link was distributed through various channels such as personal networks, organizational websites, and social media groups of national and international clubs. The sample size was determined using the single population proportion formula, based on a 13.8% prevalence of exclusive breastfeeding among employed mothers in Nepal from a prior study [18]. Using a 95% confidence interval and a 5% margin of error, the sample size was determined accordingly.


$$n=\;\frac{z^{2}P(1-P)}{d^{2}}$$

= 183 + 18 (10% non-response rate)

= 201

where, z = constant which is 1.96 at a 95% confidence interval (CI)

P (proportion) = 0.138

1-P = 0.862

d = allowable error, i.e., 5% = 0.05

Therefore, the study sample size for the study was about 201 employed mothers.

### Instrumentation for selected data collection method

A structured, web-based questionnaire was developed based on guidelines from the World Alliance for Breastfeeding Action and similar studies [19,20]. The questionnaire included socio-demographic variables, breastfeeding behaviors, and work-related factors such as: duration and type of maternity leave, presence of lactation rooms, task adjustment, availability of flexible schedules, supervisor gender, organizational communication on support, and access to breastfeeding-friendly infrastructure. EBF was assessed by asking mothers whether they provided only breast milk (without water or food) for the first six months. It was pretested among 20 employed mothers to ensure validity and clarity. The questionnaire was available in both English and Nepali, with automated checks integrated into the design, such as mandatory fields, logical branching, and error prompts, to ensure the completeness and accuracy of responses. Data collection took place from May 8, 2023, to June 7, 2023. Responses were automatically recorded in an electronic format upon submission, and daily checks were performed to ensure data accuracy. After data collection, the dataset was transferred to Microsoft Excel, then imported into SPSS version 16 for analysis. Descriptive statistics followed by bivariate logistic regression analysis was performed to calculate odds ratios (OR) for association with EBF to determine the significant predictors. A p-value of ≤0.05 was considered statistically significant.

### Operational definition of key variables

**Table:** 

Variable	Survey Measurement	Context in Nepal
**Type of Employment**	Permanent/Temporary	**Permanent**: Full-time, formally contracted, with benefits (e.g., leave, provident fund, insurance). **Temporary**: Short-term contracts with limited or no benefits. Common in NGOs, schools, and health facilities.
**Employment Sector**	Government/Private/NGO/Health/Education, etc.	Government jobs are highly regulated with mandated benefits. Private and NGO sectors may vary in policy adherence. Informal sector often excluded from protections.
**Work Arrangement**	Fixed location/Frequent travel	Some jobs require outstation travel (e.g., health mobilizers, sales), reducing workplace-based support options like breastfeeding rooms.
**Working Hours**	≤ 7 hours/ > 7 hours per day	Standard workday is 8 hours. Long work hours reduce time for direct breastfeeding or expressing milk. Breaks are not legally guaranteed.
**Maternity Leave Duration**	< 3 months/ ≥ 3 months	By law, 14 weeks (98 days) leave is required, but **only 60 days are paid**. Any leave beyond this is unpaid unless voluntarily offered by employer.
**Task Adjustment/ Lighter Workload**	Yes/No	Not legally mandated. Provided at employer discretion. More common in female-friendly environments like health or education sectors.
**Flexible Work Hours**	Breaks every 1–2 hours/Shorter day/Work from home	No national policy mandates these supports. Flexibility depends entirely on employer. Mostly limited to elite private or NGO settings.
**Information About Workplace Support**	Yes/No	Many organizations do not disseminate clear HR policies on breastfeeding support. Lack of awareness persists even when support exists.

### Data analysis

Data were analyzed using IBM SPSS Statistics version 16. Descriptive statistics including frequencies, percentages, means, and standard deviations were calculated to summarize the participants’ socio-demographic and occupational characteristics. Bivariate logistic regression analysis was performed to examine the association between exclusive breastfeeding (EBF) and independent variables such as employment characteristics and workplace support measures. Crude odds ratios (COR) with 95% confidence intervals (CI) were calculated. A p-value of ≤ 0.05 was considered statistically significant.

## Results

Table 1 explains the socio-demographic characteristics of the respondents. The respondents’ mean age (±SD) was 30.9 ± 3.7 years. The majority of the respondents were Hindu followers, and 48.0% of the respondents were affiliated with the Brahmin/Chhetri ethnic group. Regarding educational background, a substantial proportion (87.6%) of respondents reported attaining a bachelor’s degree or higher education.

**Table 1:** Socio-demographic characteristics of respondents.

Serial Number	Variables	Frequency	Percentage
1	**Age of mother**		
	< 30 years	110	54.7
	≥ 30 years	91	45.3
Median = 30, QD = 30.1, Minimum = 21, Maximum = 43			
2	**Religion**		
	Hindu	183	91.0
	Buddhist	12	6.0
	Christian	2	1.0
	Muslim	2	1.0
	Kirat	2	1.0
3	**Ethnicity**		
	Brahmin/Chhetri	97	48.3
	Others	104	51.7
4	**Level of education**		
	Diploma and below	25	12.5
	Undergraduate	88	43.8
	Graduate and above	88	43.8
5	**Province**		
	Koshi province	25	12.4
	Madesh province	39	19.4
	Bagmati province	102	50.7
	Gandaki Province	8	4.0
	Lumbini Province	11	5.5
	Karnali province	6	3.0
	Sudurpashchim province	10	5.0
6	**Marital Status**		
	Unmarried	0	0.0
	Married	200	99.5
	Separated	1	0.5
7	**Type of family**		
	Nuclear	81	40.3
	Joint	120	59.7
8	**Family economic level**		
	upto NPR 98000 per month	79	39.3
	> NPR 98000 per month	122	60.7
Median = 100000, QD = 113750, Minimum = 10000, Maximum = 150000			
9	**Number of Children**		
	1 child	148	73.6
	> 1 child	53	26.4

Table 2 describes the occupational history of the respondents. Majorities (93.5%) of the respondents were employed within national organizations, and about 30.8% indicated working in government sector. More than 70% of the respondents had a fixed job arrangement while one-fourth had to frequently travel in their jobs. Additionally, the average time taken for commuting to work was recorded 25 minutes.

**Table 2:** Occupation history of respondents.

Serial Number	Variables	Frequency	Percentage
1	**Organization Level**		
	National level organization	188	93.5
	International organization	13	6.5
2	**Job site**		
	Bank	37	18.4
	School	19	9.4
	University/College	16	8.0
	Hospital/ Health Institute	45	22.4
	Private company	35	17.4
	I/NGOs	33	16.4
	Government office	16	8.0
3	**Job Type**		
	Government	62	30.8
	Private	139	69.2
4	**Type of Employment**		
	Permanent	89	44.3
	Temporary	112	55.7
5	**Work Arrangement**		
	Fixed	144	71.6
	Outstation	6	3.0
	Fix with frequent visit	51	25.4
6	**Work Period**		
	<5 years	144	71.6
	5 years or more	57	28.4
7	**Working Hours per Day**		
	Up to 7 hours	111	55.2
	> 7 hours	90	44.8
Median = 7, QD = 7.5, Minimum = 04, Maximum = 12			
8	**Work Distance from Home**		
	Up to 20 minutes	109	54.2
	> 20 minutes	92	45.8
Median = 20, QD = 22.5, Minimum = 02, Maximum = 60			

Note: Permanent jobs referred to those with formal contracts and social security benefits, while temporary jobs included contract-based, part-time, or informal arrangements. Formal employment is typically associated with entitlement to maternity protection, though enforcement varies.

The majority (71.6%) initiated breastfeeding within the first hour after childbirth, emphasizing early breastfeeding initiation. However, exclusive breastfeeding was less common, with only 37.8% practicing it. Among those not exclusively breastfeeding, a significant proportion (76.4%) cited the need to return to work as the primary reason, indicating the challenge of balancing work and breastfeeding. Other reasons included issues related to breast milk production and the preferences of the child. Presently, 71.1% of respondents reported currently breastfeeding their child (Table 3).

**Table 3:** Breastfeeding practices among working mothers.

Serial number	Variables	Frequency	Percentage
1	**Breastfed child within 1 hour after delivery**		
	Yes	144	71.6
	No	57	28.4
2	**Exclusive breastfeeding practice (upto 6 months)**		
	Yes	76	37.8
	No	125	62.2
3	**Reason for not breastfeeding exclusively (n = 125)**		
	Return to work	97	76.4
	Time management for breastfeeding	4	3.1
	Lack of breast milk production	23	18.1
	Breast milk is not preferred by the child	2	1.6
	Knowledge on breastfeeding	1	0.8
4	**Are you currently breastfeeding your child**		
	Yes	143	71.1
	No	58	28.9

Table 4: Workplace support explains the workplace support obtained by the respondent in their working organization. The majority of respondents (94.0%) indicated that their organization had breastfeeding policies, with maternity leave being a key aspect (98.9%). Maternity leave was prevalent (93.0%), predominantly lasting three months and above (70.6%).

**Table 4:** Workplace support.

S.N	Variables	Frequency	Percentage
1	**Breastfeeding policies in the organization**		
	Yes	189	94.0
	No	12	6.0
2	**Breastfeeding policies (n = 189)**		
	Maternity leave	186	98.9
	Paternal Leave	51	27.1
	Less workload	16	8.5
	Feasible time period	45	23.9
	Separate room for breastfeeding	37	19.7
	Work from home	2	1.1
	Extra financial support	10	5.3
3	**Practice of breastfeeding support in the Workplace**		
	Maternity leave	187	96.4
	Lighter job during lactation	103	53.1
	Flexible schedule	123	63.4
	Separate room for breastfeeding	38	19.6
	Separate refrigerator to store milk	14	7.2
	Breast pump to express milk	8	4.1
4	**Maternity leave**		
	Yes	187	93.0
	No	14	7.0
5	**Period of maternity leave (n = 187)**		
	< 3 months	55	29.4
	≥ 3 months	132	70.6
Median = 03, QD = 30.1, Minimum = 01, Maximum = 06			
6	**Task adjustment or lighter workload**		
	Yes	103	51.2
	No	98	48.8
7	**Flexible working hours**		
	Break in every 1 hour	25	17.7
	Break in every 2 hours	45	31.9
	Lesser working hours	67	47.5
	Work from home	3	2.1
	Unpaid leave	1	0.7
8	**Separate room for breastfeeding**		
	Yes	38	18.9
	No	163	81.1
9	**Separate refrigerator for milk storage**		
	Yes	14	7.0
	No	187	93.0
10	**Breast pump for milk storage**		
	Yes	8	4.0
	No	193	96.0
11	**Information received about workplace support**		
	Yes	65	32.3
	No	136	67.7
12	**Supervisor**		
	Male	118	58.7
	Female	83	41.3

Table 5 shows the significant association between breastfeeding practices with different independent variables such as working period, distance between home and workplace, task adjustment during breastfeeding, flexible time, separate room for breastfeeding, and information sharing by the organization regarding the organization support for exclusive breastfeeding practice in the workplace.

**Table 5:** Association of breastfeeding practice and other variables.

Factor	Exclusive breastfeeding practices (N = 201)		Odds Ratio (95% CI)	p-value
	No (%)	Yes (%)		
**Age group of mother**				
≤ 30	69 (62.7%)	41 (37.3%)	Ref	
> 30	56 (61.5%)	35 (38.5%)	1.05 (0.59–1.86)	0.863
**Ethnicity**				
Brahmin/Chhetri	61 (62.9%)	36 (37.1%)	Ref	
Others	64 (61.5%)	40 (38.5%)	1.06 (0.60–1.87)	0.844
**Type of family**				
Nuclear	45 (55.6%	36 (44.4%)	Ref	
Joint	80 (66.7%)	40 (33.3%)	0.62 (0.35–1.12)	0.111
**Working hours per day**				
≤ 7 hrs	69 (62.2%)	42 (37.8%)	Ref	
> 7 hrs	56 (62.2%)	34 (37.8%)	0.99 (0.56–1.77)	0.993
**Number of family members**				
≤ 5	68 (61.3%)	43 (38.7%)	Ref	
> 5	57 (63.3%)	33 (36.7%)	0.92 (0.52–1.63)	0.763
**Family monthly income (Rs)**				
≤ 98000	47 (59.5%)	32 (40.5%)	Ref	
> 98000	78 (63.9%)	44 (36.1%)	0.83 (0.46–1.48)	0.526
**Number of children**				
One	93 (62.8%)	55 (37.2%)	Ref	
More than one	32 (60.4%)	21 (39.6%)	1.11 (0.58–2.11)	0.751
**Working period**				
< 5 years	98 (68.1%)	46 (31.9%)	Ref	
≥ 5 years	27 (47.4%)	30 (52.6%)	2.36 (1.26–4.43)	0.006
**Job type**				
Government	35 (56.5%)	27 (43.5%)	Ref	
Private	90 (64.7%)	49 (35.3%)	0.71 (0.38–1.30)	0.263
**Type of employment**				
Permanent	58 (65.2%)	31 (34.8%)	Ref	
Temporary	67 (59.8%)	45 (40.2%)	1.25 (0.71–2.24)	0.437
**Distance between home and workplace (minutes)**				
≤ 20 min	58 (53.2%)	51 (46.8%)	Ref	
>20 min	67 (72.8%)	25 (27.2%)	0.42 (0.23–0.77)	0.004
**Maternity leave**				
No	10 (71.4%)	4 (28.6%)	Ref	
Yes	115 (61.5%)	72 (38.5%)	1.56 (0.47–5.18)	0.46
**Maternity leave period**				
< 3 months	38 (70.4%)	16 (29.6%)	Ref	
≥ 3 months	75 (56.8%)	57 (43.2%)	1.80 (0.92–3.55)	0.086
**Task adjustment or lighter job**				
No	77 (78.6%)	21 (21.4%)	Ref	
Yes	48 (46.6%)	55 (53.4%)	4.20 (2.26–7.80)	<0.001
**Separate room for breastfeeding**				
No	118(58.7%)	45 (22.4%)	Ref	
Yes	7 (18.4%)	31 (81.6%)	11.6 (4.7–28.2)	<0.001
**Information regarding maternity service**				
No	97 (71.3%)	39 (28.7%)	Ref	
Yes	28 (43.1%)	37 (56.9%)	3.28 (1.77–6.08)	<0.001
**Supervisor**				
Male	75 (63.6%)	43 (36.4%)	Ref	
Female	50 (60.2%)	33 (39.8%)	1.15 (0.65–2.05)	0.633

## Discussion

This study explored the critical challenges faced by employed mothers in Nepal in maintaining exclusive breastfeeding (EBF) and highlights the role of workplace support in promoting this practice. The prevalence of EBF among working mothers in this study was 37.8%, which is higher than Dhaka, Bangladesh (29%) [21] and Kathmandu, Nepal (13.8%) [21] however, much lower than the national average of 56% as reported by the Nepal Demographic and Health Survey (NDHS) 2022 [13].

The fundamental contradiction in Nepal’s maternal health policy becomes apparent when examining the disconnect between WHO recommendations and actual support mechanisms. While Nepal’s legal framework ensures 98 days (14 weeks) of maternity leave, of which 60 days is fully paid, this creates an untenable situation where the government expects mothers to maintain six months of exclusive breastfeeding with less than three months of financial support. Neighboring countries like India offer more extensive provisions, with 26 weeks (6 months) of fully paid leave [20]. Similarly, Vietnam mandates 6 months of paid leave and enforce workplace breastfeeding supports [22].

This policy gap raises critical questions about how governments can realistically expect optimal infant feeding practices when the structural support systems are fundamentally inadequate. The expectation of six months of EBF becomes particularly problematic when mothers are forced to return to work after only 8.5 weeks of paid leave, creating an impossible choice between economic survival and optimal child nutrition. The remaining 5.5 weeks of unpaid leave are often inaccessible for women in low-income employment, who constitute the majority of Nepal’s female workforce.

Despite Nepal’s policy alignment with the ILO’s minimum standard of 14 weeks of maternity leave, only 60 days (approximately 8.5 weeks) of this period is legally mandated as fully paid, while the remaining 5.5 weeks are unpaid unless voluntarily covered by employers. This distinction between the duration of leave and the paid portion is critical, as unpaid leave is often inaccessible for women in low-income employment. Consequently, women may have to return to work after only 8.5 weeks, limiting their ability to continue exclusive breastfeeding. As a result, although the duration meets international standards on paper, its partial remuneration may undermine its practical impact. Nepal’s exclusive breastfeeding (EBF) rates among employed mothers remain lower than the national average, suggesting that the duration of leave alone is insufficient without additional workplace accommodations (e.g., lactation rooms, task adjustments, and flexible hours).

Nepal’s childcare ecosystem remains predominantly informal and community-based, creating significant challenges for working mothers attempting to maintain EBF. Over half of the respondents in the study held temporary positions, reflecting Nepal’s high rate of vulnerable employment. Notably, 78% of employed women in Nepal work in informal sectors [1], where legal protections are often unenforced, further limiting EBF sustainability. This informal employment structure is mirrored by equally informal childcare arrangements, typically relying on extended family networks, particularly grandmothers and female relatives, rather than structured institutional support.

The reliance on informal childcare creates several barriers to EBF maintenance. Family caregivers, while well-intentioned, often lack knowledge about optimal feeding practices and may introduce complementary foods earlier than recommended. Additionally, the geographic dispersion of extended families in modern Nepal means many mothers lack access to traditional support networks, leaving them without viable childcare alternatives when returning to work.

The formal childcare system in Nepal remains severely underdeveloped. While the government has initiated Early Childhood Development (ECD) programs, these primarily focus on children aged 3–5 years rather than infants requiring EBF support [23]. Workplace crèches, mandated under various labor laws, remain largely theoretical rather than practical, with only minimal enforcement and implementation across sectors.

Private childcare facilities are concentrated in urban areas and remain financially inaccessible to most working mothers [23]. Non-governmental organizations have attempted to fill this gap through community-based childcare initiatives, but these efforts remain fragmented and lack systematic coverage [24]. The absence of standardized quality assurance mechanisms further compromises the reliability of available services.

The EBF prevalence in this study falls short of both national and international targets. The World Health Organization (WHO) aims to achieve at least 50% EBF by 2025 [25] as part of its Global Nutrition Targets and 70% by 2030 under the Nutrition Strategy 2030 [26]. EBF is critical for infant survival, offering protection against malnutrition, infections, and developmental delays while imparting stronger immune systems and cognitive development [27]. Globally, countries with stronger breastfeeding policies and enforcement mechanisms, such as Norway and Sweden, report EBF rates exceeding 70% [28]. The disparities observed in Nepal emphasize the need for systemic changes to workplace policies and enforcement to align with these global standards.

Countries that have successfully integrated childcare systems with breastfeeding support demonstrate the feasibility of comprehensive approaches. Nordic countries combine extended parental leave with accessible, high-quality childcare that accommodates breastfeeding mothers. These systems recognize that supporting EBF requires not just individual accommodation but systemic restructuring of work-family balance policies.

Access to dedicated breastfeeding rooms emerged as a critical factor, with mothers who had such access being over 11 times more likely to practice EBF. This finding aligns with previous systematic review showing that breastfeeding-friendly environments, including lactation rooms, greatly improve breastfeeding rates among working women [29] and is consistent with the ILO Maternity Protection Convention (C183), which mandates that employers provide safe, hygienic, and private breastfeeding facilities for working mothers [30]. Despite these international guidelines, only 18.9% of respondents in this study reported access to such rooms. This reflects the weak implementation of maternity protection policies in Nepal and highlights an urgent need to institutionalize the ILO’s recommendations at the workplace level.

The absence of workplace childcare facilities compounds these challenges. Even when breastfeeding rooms are available, mothers face the additional burden of arranging childcare that accommodates frequent nursing breaks or pump-and-store routines. Without on-site childcare or nearby facilities that support breastfeeding mothers, even well-intentioned workplace accommodations remain insufficient.

Comparatively, countries that have fully adopted these guidelines, such as Finland and Denmark, have demonstrated remarkable success in achieving high EBF rates [31]. The absence of breastfeeding rooms in Nepalese workplaces may also reflect infrastructural limitations, insufficient awareness among employers, and inadequate monitoring by regulatory authorities.

Flexible work arrangements and task adjustments are strongly associated with increased EBF rates [30], as observed in this study where mothers with task adjustments were four times more likely to breastfeed exclusively. This demonstrates the importance of workplace flexibility in supporting breastfeeding mothers, as those with a manageable workload are more likely to maintain EBF [32]. The ILO Maternity Protection Recommendation (R191) Article 10 explicitly supports the provision of paid breastfeeding breaks or a reduction in working hours for lactating mothers without any loss of pay [33]. However, only 51.2% of mothers in this study reported receiving such accommodations, highlighting significant gaps in policy enforcement.

The integration of flexible work arrangements with childcare support becomes crucial when considering the extended duration of EBF. Countries like Germany and Australia, which offer flexible work hours and enforce mandatory breastfeeding breaks, have reported significant improvements in maternal and child health outcomes. Evidence from South Asia further emphasizes the importance of workplace flexibility in enabling mothers to continue breastfeeding, especially in cultures where employment is seen as conflicting with caregiving [34,35].

Employment tenure played a crucial role, wherein mothers who had been employed for five years or more were twice as likely to practice EBF compared to those with shorter tenure. This suggests that more experienced employees may have better work-life balance or more supportive work environments, enabling them to continue breastfeeding [36]. Unequal access to breastfeeding support based on tenure highlights the need for formalized policies that ensure equitable access for all employees, including those in temporary or contractual positions.

In urban areas, long commutes often deprive mothers of the opportunity to breastfeed during breaks, creating a structural barrier to achieving optimal feeding practices. Mothers whose workplace was within 20 minutes of their home were more likely to exclusively breastfeed than those with a longer commute. Similar findings have been reported from China [32] and Kenya [37] where women living closer to workplace could manage time to breastfeed during breaks. This aligns with the UNICEF’s Family-Friendly Workplace Initiative, which recognizes workplace proximity and telecommuting as critical enablers of EBF [38].

The geographic dispersion of childcare options further complicates these challenges. Even when formal childcare exists, its location relative to both home and workplace significantly impacts its utility for supporting EBF. The absence of integrated planning between childcare provision, workplace policies, and residential development creates additional barriers that individual mothers cannot overcome through personal effort alone.

Information dissemination about workplace breastfeeding support was another significant factor. Mothers who received information about breastfeeding support services were three times more likely to practice EBF than those who did not. This highlights the need for better communication from employers regarding available support, as knowledge alone can increase the likelihood of breastfeeding continuation [36]. The ILO recommends that employers actively disseminate information on maternity protection and breastfeeding rights through workshops, orientations, and policy handbooks [33]. In Nepal, these strategies remain underutilized, as evidenced by the limited awareness reported in this study.

The information gap extends beyond workplace policies to include knowledge about available childcare options and their compatibility with breastfeeding goals. Many mothers remain unaware of their rights under existing legislation or available support services, highlighting the need for comprehensive information systems that bridge formal policy and practical implementation.

Despite these findings, some factors such as the age of the mother, ethnicity, type of family, working hours, and family income were not significantly associated with EBF in this study. This contrasts with some previous studies that found associations between these socio-demographic factors and breastfeeding practice [36]. However, this study’s findings reinforce the idea that workplace support, rather than demographic factors, plays a critical role in enabling working mothers to continue EBF.

The evidence suggests that achieving optimal EBF rates requires coordinated policy reform across multiple domains: extended paid maternity leave that aligns with WHO recommendations, development of formal childcare infrastructure that supports breastfeeding, enforcement of workplace accommodation requirements, and integration of childcare planning with broader urban and employment policy.

The respondents predominantly represented white-collar workers, limiting generalizability. Biases introduced by non-probability sampling and the overrepresentation of white-collar workers highlight the need for more inclusive research. Addressing these limitations and implementing breastfeeding-friendly policies across diverse occupational sectors, particularly focusing on the informal sector where most women work, can significantly enhance maternal and child health outcomes in Nepal.

## Conclusion

This study highlights the critical role of workplace accommodations, including extended maternity leave, flexible work hours, task adjustments, and access to breastfeeding rooms, in supporting exclusive breastfeeding (EBF) for the first six months. The study’s findings align with global research that calls for enhanced workplace policies to support breastfeeding, including longer paid maternity leave and dedicated lactation space. However, the fundamental contradiction between expecting six months of EBF while providing only 8.5 weeks of paid leave exposes the inadequacy of current policy frameworks.

Given the medical benefits of EBF for newborn children, and rising participation of women into the workforce, there is an urgent need for comprehensive policy reform that recognizes the interconnected nature of maternal employment, childcare systems, and infant nutrition. This includes extending paid maternity leave to six months to align with EBF recommendations; developing formal childcare infrastructure that accommodates breastfeeding mothers; ensuring workplace breastfeeding accommodations like lactation rooms and flexible hours; and strengthening the enforcement of these policies, particularly for informal sector workers.

Without addressing these systemic gaps, governments cannot reasonably expect to achieve optimal breastfeeding outcomes while simultaneously promoting women’s economic participation. The solution requires not just policy adjustment but fundamental restructuring of how societies support working mothers through the integration of employment, childcare, and health policies. Only through such comprehensive approaches can Nepal and similar countries hope to meet both their maternal employment goals and child health objectives.

## Supporting information

S1 Data Dataset in SAV format (SPSS file) containing raw data used for statistical analysis in the study on breastfeeding and workplace support in Nepal. (SAV)
